# Deepithelialized punched tissue for buccal soft tissue augmentation in implant placement

**DOI:** 10.1093/jscr/rjaf909

**Published:** 2025-11-14

**Authors:** Dler Ali Khursheed

**Affiliations:** Department of Periodontics, College of Dentistry, University of Sulaimani, Madame Mitterrand Street, Sulaymaniyah 46001, Kurdistan Region, Iraq

**Keywords:** dental implants, soft tissue augmentation, tissue punch technique, minimally invasive surgery, connective tissue graft

## Abstract

Adequate buccal soft tissue thickness is essential for long-term implant stability and aesthetics. Conventional augmentation with palatal connective tissue grafts is effective but increases morbidity and surgical time. This report presents a novel technique in which keratinized mucosa, routinely removed during guided implant placement, was preserved, deepithelialized, and reused as an autogenous graft. Two cases were treated: one with delayed loading and one with immediate loading. In both, the graft was placed into a buccal pouch and stabilized with a healing abutment. Clinical and radiographic follow-up showed uneventful healing, improved buccal soft tissue thickness, and stable peri-implant bone levels at 6 months. This technique highlights the feasibility of reusing discarded tissue as a donor-site-free grafting material within guided workflows.

## Introduction

Adequate buccal soft tissue thickness is critical for peri-implant health, stability, and aesthetics, whereas thin mucosa predisposes to bone loss, recession, and compromised outcomes [1–3]. Soft tissue augmentation with palatal connective tissue grafts remains effective but increases morbidity, surgical time, and donor-site complications [4]. The tissue punch technique, frequently used in guided implant placement, is minimally invasive and preserves vascular supply, though the excised mucosal disc is usually discarded [4–6]. This tissue provides a viable connective tissue matrix capable of supporting peri-implant regeneration [7]. It is composed of the epithelium, and the subepithelial connective tissue with the lamina propria and the submucosa [4].

This report describes a novel approach that reuses deepithelialized punched mucosa to augment buccal soft tissue at the implant site, offering a biologically sound, donor-site-free, and compatible with digital implant placement workflow.

## Case presentation

Two male patients were treated using this technique. The first, aged 40, received a delayed loading implant restored at 2 months. The second, aged 35, underwent immediate loading with crown delivery at 48 h (Table 1). Both were systemically healthy and nonsmokers. The first case demonstrates the graft’s influence on buccal tissue volume, while the second shows feasibility in immediate loading with definitive crown restoration.

**Table 1:** Case details

**Case**	**Age (years)**	**Implant site**	**Implant size (mm)**	**Abutment gingival height (mm)**	**Loading protocol**
1	40	#46	4.5 × 11.5	1	Delayed
2	35	#36	5 × 13	2	Immediate

**Figure 1:**
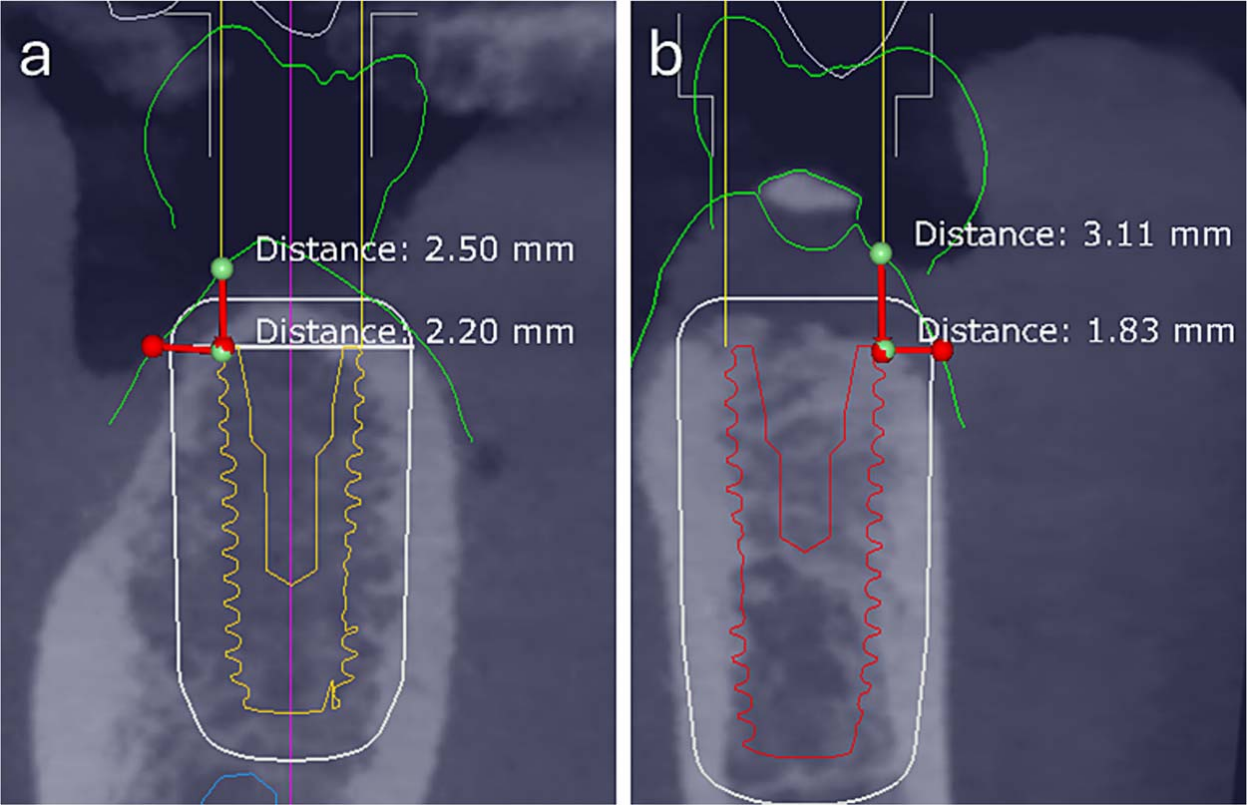
Preoperative digital assessment of buccal soft and hard tissue thickness using cross-sectional CBCT planning views. The measured distances represent the combined buccal bone and mucosal thickness from the buccal crest of the planned implant to the external surface of the mucosa. (a) Case 1: Total mid-buccal tissue thickness ≈ 2.20 mm; vertical thickness ≈ 2.50 mm. (b) Case 2: Total mid-buccal tissue thickness ≈ 1.83 mm; vertical thickness ≈ 3.11 mm.

Clinical and radiographic assessments included intraoral scanning and CBCT. Guided surgeries were digitally planned using Exoplan 3.1 (exocad, Germany), and the surgical guides were printed [8]. In the first and second cases, the total buccal tissue complex (bone + mucosa) measured ~2.20 and 1.83 mm, respectively, when assessed from the most buccal implant crest to the external mucosal surface (Fig. 1).

### Surgical procedure

Patients rinsed preoperatively with 0.12% chlorhexidine and 0.05% CPC (Perio·Aid®, Spain). Guides were cold-sterilized and the inner surfaces coated with chlorhexidine–hyaluronic acid gel.

A 4.5 mm tissue punch (OneGuide System, Osstem Implant, Korea) was used for access; ridge convexity required freehand completion. The harvested keratinized mucosa was detached with tunnelling instruments and preserved in saline. Implant osteotomy and placement followed the manufacturer’s protocol.

**Figure 2:**
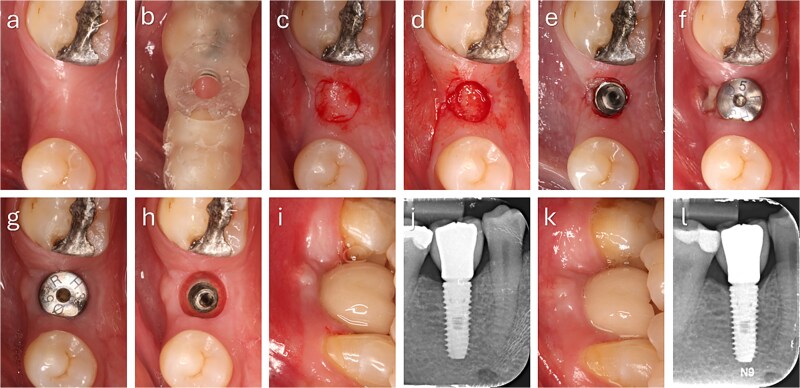
Step-by-step clinical sequence of buccal soft tissue augmentation with deepithelialized punched tissue during delayed loading implant placement. The graft demonstrated stable integration and enhanced buccal mucosal volume at follow-up. (a) Preoperative occlusal view. (b) Surgical guide in position. (c) Exposure of keratinized tissue. (d) Removal of punched tissue. (e) Implant placement. (f) Graft positioned in buccal pouch. (g) Two-month follow-up showing improved buccal contour. (h) Mucosa after tissue former removal. (i) Final crown at two months. (j) Radiograph at two months. (k) Six-month follow-up with stable peri-implant mucosa. (l) Radiograph at six months showing marginal bone remodeling.

**Figure 3:**
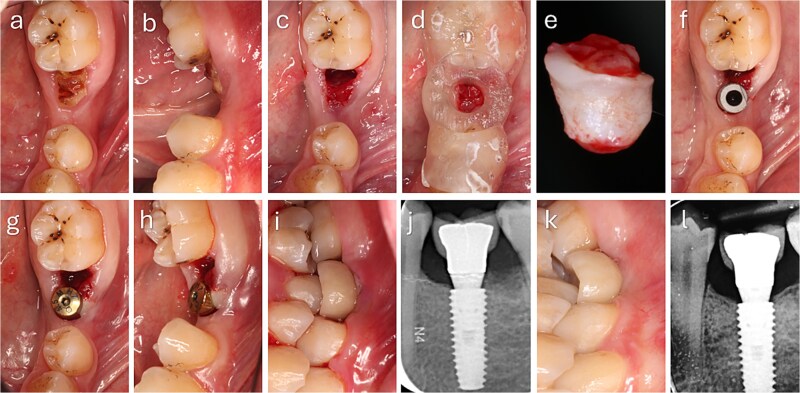
Clinical sequence of buccal augmentation with deepithelialized punched tissue during immediate implant loading. The graft was incorporated successfully, with stable peri-implant tissues at six months. (a, b) Preoperative occlusal and lateral views with atrophic ridge and retained root. (c) Occlusal view after extraction. (d) Surgical guide in position. (e) Harvested keratinized punched tissue. (f) Scan body placement. (g, h) Graft insertion and tissue former placement. (i) Crown delivered at 48 hours. (j) Radiograph after crown placement. (k) Six-month follow-up with healthy peri-implant mucosa. (l) Radiograph at 6 months showing no bone loss.

The punched graft was stabilized with corn tweezers and deepithelialized using a #15c blade (Supplementary Video S1). A buccal pouch was created with a Kirkland knife and tunnelling instruments, and the graft inserted with orientation adapted to thickness: in thin grafts (Fig. 2), the connective surface faced the implant; in thicker grafts (Fig. 3), it was oriented apically. A healing abutment coated with chlorhexidine–hyaluronic gel supported graft stabilization without sutures. A definitive crown was placed after 48 hours of implant placement in immediate loaded case.

**Figure 4:**
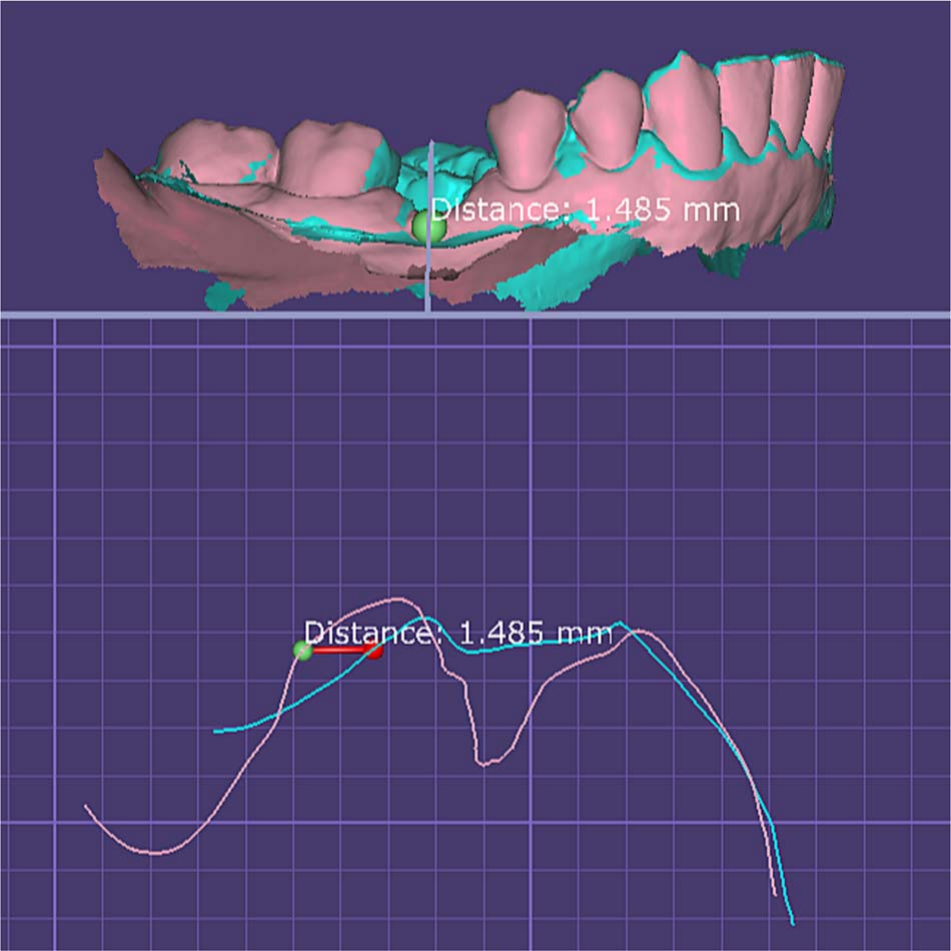
Quantitative digital evaluation of buccal mucosal thickness before and after graft healing. Superimposed STL models show the pre-grafting surface and the 2-month postoperative surface after alignment in *DentalCAD 3.1 (exocad, Germany)*. Linear measurement taken at the widest buccal contour demonstrates an increase in mucosal thickness of ~1.49 mm between the two time points.

For immediate loading, digital scans were obtained before graft placement to ensure accuracy; in delayed loading, scans were performed at two months, confirming improved buccal tissue thickness and stability prior to definitive restoration (Fig. 4).

## Discussion

These cases demonstrate that discarded punched mucosa can be reused as a deepithelialized autogenous graft to enhance buccal peri-implant tissues. The approach is minimally invasive, donor-site-free, and integrates smoothly into guided workflows.

Augmentation at the buccal aspect is clinically significant, as this site is most prone to recession and aesthetic compromise [9, 10]. Evidence indicates that a mucosal thickness ≥2 mm reduces marginal bone loss, while thinner tissues are more vulnerable [11]. Recent systematic reviews confirm that autogenous grafts provide superior stability compared with substitutes [4, 12]. Unlike guided bone regeneration (GBR) procedures that primarily target hard tissue augmentation and often require membrane and bone substitute placement [13], the described technique focuses on enhancing the peri-implant soft tissue envelope using autogenous *in situ* tissue. It is particularly indicated when adequate ridge width exists and soft tissue deficiency is the main concern. The method complements, rather than replaces, GBR and can be applied in situations where bone volume is already sufficient.

In this proof-of-concept, both cases showed improved buccal soft tissue thickness, stable mucosa, and satisfactory aesthetics at 6 months, without complications. While peri-implant tissue thickness is a key determinant, implant–abutment connection and prosthetic design also contribute to marginal bone stability [14, 15]. Patient preference to avoid palatal harvesting further supports the relevance of this method.

The technique is limited by graft size, lack of volumetric measurements, and short follow-up. Larger, controlled studies with patient-reported outcomes are needed to validate predictability.

## Conclusion

Reusing deepithelialized punched mucosa provides a simple, biologically sound, and minimally invasive method for buccal soft tissue augmentation. It avoids palatal donor sites, reduces morbidity, and may serve as a practical adjunct in implant therapy.

## Supplementary Material

Video_1_rjaf909

## Clinical and surgical implications

This technique reuses tissue normally discarded, enhances buccal peri-implant thickness without a secondary donor site, and supports the ≥2 mm mucosal threshold for long-term stability and aesthetics.
